# IR–UV Ion Dip Spectroscopy of Capped Phenylated Polyalanines in the Gas Phase

**DOI:** 10.1021/acs.jpca.5c05537

**Published:** 2026-01-15

**Authors:** Åke Andersson, Piero Ferrari, Imre Bakó, Vitali Zhaunerchyk

**Affiliations:** † Department of Physics, 3570 University of Gothenburg, 41296 Gothenburg, Sweden; ‡ HFML-FELIX, 6029 Radboud University, Nijmegen 6525 ED, The Netherlands; § MTA Kémiai Kutatóközpont Szerkezeti Kémiai Intézet, Pusztaszeri út 59-67, 1025 Budapest, Magyarország

## Abstract

The gas-phase structures of capped phenylated polyalanine peptides, Ac-Ala-Ala-Phe-Ala-NH_2_ (AAFA) and Ac-Ala-Ala-Phe-Ala-Ala-NH_2_ (AAFAA), were investigated using conformer-selective IR–UV ion dip spectroscopy employing the IR light of the FELIX free electron laser. IR absorption spectra were measured in the wide 300–1900 cm^–1^ range and additionally in the 3200–3600 cm^–1^ region, complemented by extensive quantum-chemical calculations. The AAFA peptide was found to adopt a single dominant conformer with a β-hairpin structure stabilized by four hydrogen bonds, whose predicted spectrum closely matches the experimental data. In contrast, no conformer of AAFAA matches the experimental spectrum, despite generating over 200,000 conformers across multiple search strategies, suggesting that the true structure was not found. Additionally, computations of the molecules with and without the phenyl group reveal an induced alteration of the conformational landscape.

## Graphical abstract

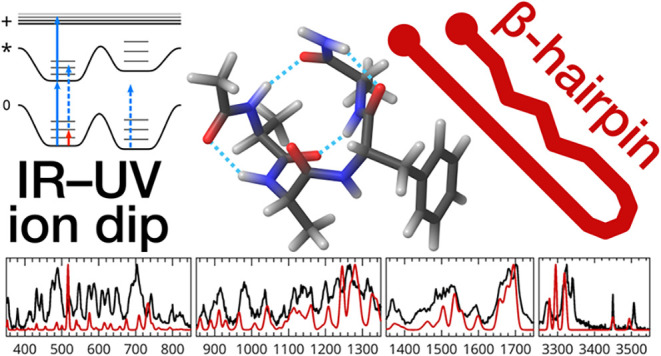

## 1 Introduction

The function of a protein is caused by its folded shape, which is stabilized by secondary structures such as helices, which is in turn determined by its primary sequence. In particular, segments of repeated alanine (Ala) are known to form helices in water.^1^ Because experiments in the gas phase better allow for measurements of intrinsic properties, the circumstances under which gas-phase polyalanine peptides form helices have been studied experimentally and theoretically.^2–17^ Two influential parameters on the structure appear to be terminal residues^16^ and peptide length.^9–11^

A simulation^16^ of gas-phase Ala_10_ showed that it fully formed a α-helix, but only partially when lysine was added to any terminus. Similar ionic peptides have been experimentally investigated and found^9–11^ to transition from globular to helical structures as the length increases, with the transition occurring between 5 and 10 residues. This is close to the typical length of polyalanine sequences in natural proteins, which is 10.^18^

Previous studies on Ala_5_ have found hints of helicity, but unfortunately they have not been fully conclusive due to congested spectra arising from multiple conformers.^2^ A possible remedy to this problem is the use of conformer-selective ion-dip spectroscopy, which requires the inclusion of a peptide unit with high UV absorption.^19^ This can be achieved by replacing one Ala unit by Phe, which contains a UV-absorbing chromophore. Moreover, using the common Ac and NH_2_ caps allows us to limit termini interactions, which seldom occur in natural proteins. This is motivated by the fact that in our study of Ala_5_, termini interactions determined the structure.^2^

In this work, we study the capped phenylated polyalanines Ac-Ala-Ala-Phe-Ala-NH_2_ (AAFA) and Ac-Ala-Ala-Phe-Ala-Ala-NH_2_ (AAFAA) using conformer-specific IR–UV spectroscopy. Similar capped phenylated polyalanines of length 2–4 have previously been studied^5,17,19,20^ and predictably found to not form helices. By comparing the structures of AAFA in this study to the previously studied^17,20^ Ac-(Ala)_3_-Phe-NH_2_ (AAAF) and Ac-(Ala)_4_-O-Bzl (AAAAb), we can estimate the importance of the phenyl substitution site. Moreover, AAFAA is interesting because it is a candidate for the shortest helical polyalanine.

## 2 Methods

### 2.1 Spectroscopic Experiment and Model

All experiments were performed in a laser desorption setup at the HFML-FELIX Laboratory, described in detail elsewhere.^21^ The species (synthesized by ProteoGenix, purity >95%) of Ac-Ala-Ala-Phe-Ala-NH_2_ (AAFA) and Ac-Ala-Ala-Phe-Ala-Ala-NH_2_ (AAFAA) were studied in the gas phase using IR–UV ion dip spectroscopy.^22^ This technique measures the IR absorption spectrum of a species via its vibrational ground state abundance using UV resonance-enhanced multiphoton ionization (REMPI), making it a conformer-sensitive technique.

First, the AAFA­[A] sample was mixed with carbon powder and pressed onto a movable graphite bar, which was inserted into the source vacuum chamber. Pulses from a 1064 nm desorption laser were used to deliver the sample to the gas phase. For the purpose of optimizing the yield from laser desorption, a high-frequency ArF excimer UV laser (193 nm) was used to ionize the molecules with nonresonant single-photon action. The ion yield was then measured with a time-of-flight mass spectrometer. The desorption laser pulse intensity and focus, and the graphite bar position were all manually tweaked to maximize ion yield.

Following, a tabletop dye laser, employing Coumarin 153 in ethanol and a doubling crystal, was used to scan the UV wavelength range of 37,313–37,736 cm^–1^ (268.00−265.00 nm) while the ion count was measured. This provided the 1 + 1 REMPI spectrum of the investigated species.

Finally, the UV dye laser was used in tandem with an IR laser, being either FELIX or a tabletop OPO laser. The UV frequency was held constant at a resonant value, while the IR laser scanned in the range of 300–1900 cm^–1^ (FELIX) or 3200–3600 cm^–1^ (OPO, LaserVision). The IR laser fired before every second UV pulse, which allowed the consecutive recording of mass spectra with and without the influence of IR interaction.

To infer the relative cross-section of IR absorption σ­(ν) from the ion yield, we use a simple, established model.^22,23^ The sample molecules enter the interaction region in the ground state. During exposure to the IR beam with frequency ν and photon flux Φ­(ν), each molecule in the ground state becomes vibrationally excited with rate Φσ. After a time τ of exposure, the ratio of ground state molecules is exp­(−Φστ). The UV pulse then ionizes a constant fraction of these, but none of the excited molecules. With these assumptions, the cross-section can be found as

1 $$\sigma \left(\nu \right)=\frac{1}{\Phi \tau }\ln\frac{P_{\mathrm{off}}}{P_{\mathrm{on}}\left(\nu \right)}$$

where *P* is the count of the unfragmented (parent) species, with the subscript telling whether the IR laser fired before. The photon fluence Φτ is an unknown normalization constant proportional to the number of photons in an IR pulse. Equation 1 therefore gives only a relative measure of the cross-section.

### 2.2 Quantum-Chemical Calculations

The conformational spaces of AAFA and AAFAA were both first investigated with two software packages: Tinker^24^ and MOPAC.^25^ Starting from four commonly occurring structures such as the α-helix and β-sheet, the basin-hopping scan program of Tinker was used with the MM3 force field to find 100,000 unique energy minima per species. All minima were further optimized with the semiempirical PM7 method implemented in MOPAC. Pairs of minima with root-sum-squared nuclei distances less than 1 Å were considered the same conformer and merged. The most stable conformer, and all others within 5 kcal/mol were selected for further analysis.

Preliminary analysis showed that the conformational space of AAFAA was not fully explored, and thus a second generation of searching was done. Starting from the most stable conformers according to PM7, 100,000 unique but not necessarily new energy minima were generated, and filtered like the rest.

The candidate conformers were then investigated with DFT as implemented in Gaussian.^26^ Optimizations and harmonic frequency analyses were done using the B3LYP functional with GD3BJ empirical dispersion and the Jun-cc-pVTZ basis set, and then repeated with seven other functionals to ensure consistency. The more expensive CBS-4M method was used for precise single-point energy calculations. The relative abundances are based on Gibbs energies at 400 K, an estimate of the laser desorption temperature.^27^ Although the molecules are subsequently cooled by supersonic jet expansion, it is assumed that the conformer distribution is constant during this process.

Afterward, a third conformational search was carried out using a third software: CREST (Conformer-Rotamer Ensemble Sampling Tool).^28,29^ The CREST program uses the iterative application of the metadynamic method to study the conformational space of the molecule under investigation. The force field used in the calculation is either the GFn2-xtb semiempirical method or the GFN-Ff force field.^30^ This complementary search did not change the outcome of AAFA, but did find a new most stable conformer of AAFAA.

Because the resulting AAFAA conformers were not satisfactory, a fourth generation of conformer searching was carried out. This one, like the first, used Tinker and MOPAC to generate 100,000 conformers, but had the phenyl group omitted during the basin hopping stage and edited back afterward. The most stable conformer produced this way had an overall energy rank of 4. Furthermore, phenyl omission gave insights into the effect of phenyl group substitution.

## 3 Results and Discussion

### 3.1 Conformational Search

Figure 1 shows selected conformers found for AAFA and AAFAA, in which the electronic energies of the conformers were finally evaluated using the CBS-4M method. The AAFA species has one conformer with energy far below (23.85 kJ mol^–1^ ∼ 2869 K) any other, which is therefore dominant. This can be attributed to its so-called β-hairpin^17,19^ structure, which allows for four hydrogen bonds. In fact, Plowright et al.^17^ remarked on the stability of this structure and found it to be common across three different tetrapeptides. The structure of AAFA is very similar to that of AAAF^17^ and chromophore-capped AAAAb,^20^ which is unsurprising considering that the phenyl group does not participate in any strong interaction.

**Figure 1:**
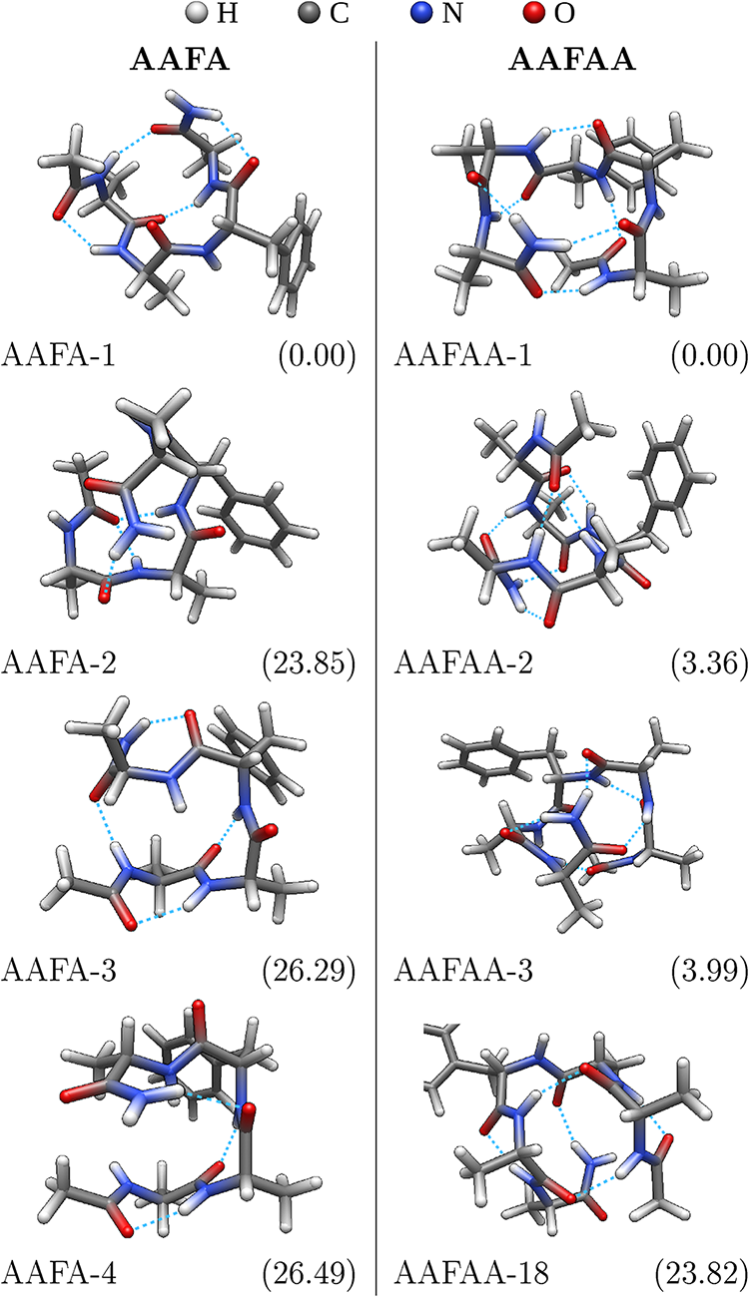
Structures of selected conformers, with possible hydrogen bonds drawn as dashed lines. The subcaptions shows CBS-4M zero-point-corrected electronic energy in kJ mol^–1^, relative to the lowest-energy conformer. The conformer names indicate the rank according to this energy. AAFAA-18 is presented because its predicted spectrum is discussed later in the text.

The AAFAA species also has one dominant conformer, but the energy difference (3.36 kJ mol^–1^ ∼ 404 K) is not as extreme. Interestingly, the top three most stable conformers are all from different generations of conformer searches, regardless if electronic or 400 K Gibbs energies are considered. This suggests that none of the searches were sufficient on their own, plausibly not even together.

In the majority of AAFAA conformers, the –NH_2_ cap participates in hydrogen bonds with both of its hydrogens. This is not realistic for a model system of proteins, because there is normally only one hydrogen. Applying a –NHCH_3_ cap to the C-terminus would resolve this issue.

When later comparing IR spectra to the experiment, none of the stable structures of AAFAA fits well. The best fit in the stretching range is with AAFAA-18, which is therefore included in Figure 1. Although its relative zero-point-corrected electronic energy (Δ*E*) is prohibitively high, the relative Gibbs energy (Δ*G*) could be different. Figure 2 explores the abundances

2 $$p=\frac{1}{Z}\exp\left(-\Delta G/k_{B}T\right);,\sum_{i}p_{i}=1$$

for the 20 conformers with lowest Δ*E*, and shows that the abundance of AAFAA-18 is less than 1% at all temperatures below 1000 K.

**Figure 2:**
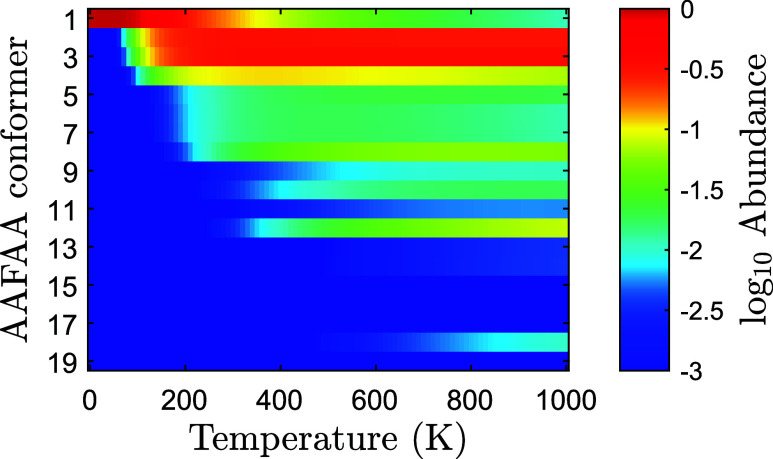
Estimated abundances for the 20 lowest Δ*E* conformers of AAFAA. Gibbs energies were calculated with the CBS-4M method for temperatures in the range of 0–1000 K.

### 3.2 UV REMPI Spectra

Figure 3 shows the experimental REMPI spectra of AAFA­[A]. The spectrum of AAFA contains three strong resonances. By contrast, the spectrum of AAFAA is faint, and only one resonance was strong enough to allow for IR–UV ion dip spectroscopy. A possible explanation for the faint spectrum of AAFAA could be that more than one conformer was present in the experiment.

**Figure 3:**
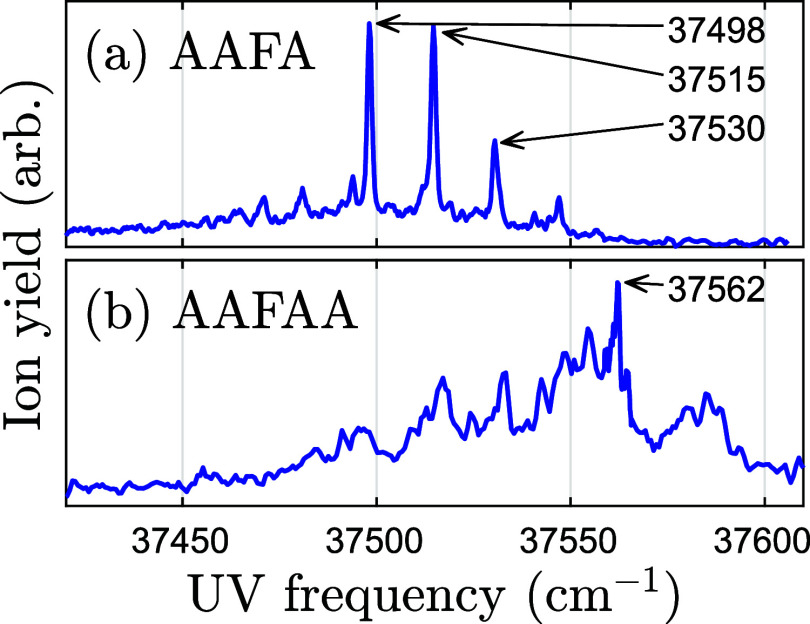
Experimental REMPI spectra of phenylated polyalanines. The blue curves show the ion yield of (a) AAFA and (b) AAFAA composited from multiple scans. The peaks later used for ion dip spectroscopy are labeled.

Four strong resonant UV frequencies of AAFA are equidistant with distance 16 ± 1 cm^–1^, with the lowest at 37,498 cm^–1^. This suggests that what we observe are transitions from the ground state to excited states differing only in the quanta of one vibrational mode. The smallest vibrational frequencies according to B3LYP-GD3BJ/Jun-cc-pVTZ (scaled with 0.980) are 17.0, 22.2, and 32.3 cm^–1^. They are reminiscent of the observed distance 16 ± 1 cm^–1^ in two ways: 17.0 ≈ 16 ± 1 cm^–1^ and 32.3 ≈ 2·16 ± 1 cm^–1^. Two assignments are therefore plausible for 37,530 cm^–1^: two quanta in the smallest vibrational frequency, or one quantum in the third smallest.

### 3.3 IR–UV Ion-Dip Spectra

The spectra of the two studied peptides are presented in separate sections.

#### 3.3.1 The Tetrapeptide AAFA

In order to verify that the three AAFA REMPI peaks are associated with the same conformer, an IR–UV ion dip scan was performed using each transition depicted in Figure 3. Figure 4 depicts the result: the three spectra are strikingly similar, therefore showing that they are associated with the same conformer, and thus can be added together into a single IR spectrum to improve statistics. This is motivated by a later result: the infrared spectra of different conformers differ significantly.

**Figure 4:**
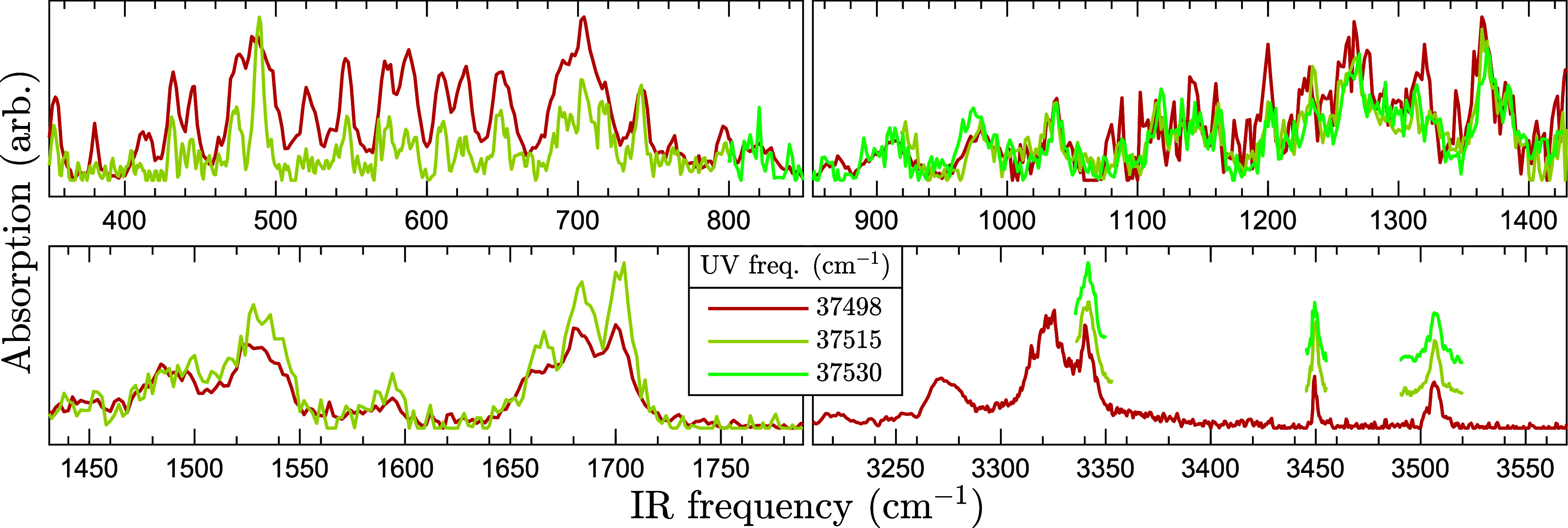
Experimental IR–UV ion dip spectra of AAFA. The solid colored curves show the absorption signal, as calculated using eq 1, with the color telling the UV frequency. Above 3000 cm^–1^, the spectra are vertically offset to reveal otherwise identical features. The three spectra are virtually indistinguishable, suggesting that they belong to the same conformer.

Figure 5 compares the run-averaged IR–UV ion dip absorption of AAFA to theoretical predictions. The spectrum of AAFA-1 matches the experiment remarkably well in the entire frequency range, unlike the other conformers. From this strong agreement, the experimental IR bands can be assigned vibrational modes. Starting at high wavenumbers and descending:

- At 3507 cm^–1^ lies the NH_2_ asymmetric stretching mode, predicted at 3494 cm^–1^. The error of 13 cm^–1^ is relatively small, as dual-scaled B3LYP has a typical error of 25 cm^–1^.^31^
- At 3450 cm^–1^ lies the only free NH stretching band, exactly where predicted.
- At 3340 and 3325 cm^–1^ there are two close IR bands of similar intensity. According to B3LYP, the NH_2_ symmetric stretching mode mixes with the NH stretching modes of the two closest nitrogens (1 and 4, counting from the N-terminus), resulting in three modes with frequencies 3323, 3318, and 3295 cm^–1^. Although it requires shifting the modes by up to 30 cm^–1^, assigning these modes to the bands most likely option.
- At 3272 cm^–1^ lies the NH stretching band belonging to nitrogen 2, which participates in an H-bond with the carbonyl of the acetate cap.

The assignment thus far has been similar to that of AAAF, which is unsurprising given their similar structure.^17^ The main difference relative to AAAAb comes from the NH_2_ group, and not the phenylation of the third residue.^20^ Continuing into the FELIX frequency range below 2000 cm^–1^:

- The broad peak at 1655–1705 cm^–1^ contains the five carbonyl stretching modes, also known as amide I. B3LYP predicts that the carbonyl from residue 2 and 4 have similar stretching frequencies 1695 ± 1 cm^–1^, which is surprising given that the 2-carbonyl is free while the 4-carbonyl accepts the strongest hydrogen bond. This coincidence results in a resolved peak, experimentally seen at 1682 cm^–1^.
- At 1594 cm^–1^ lies a single NH_2_ scissoring band, predicted at 1599 cm^–1^.
- The wide band at 1475–1565 cm^–1^ contains the four amide II modes, meaning in-plane bending of NH. According to B3LYP the frequency should vary with the type of H-bond: 1554 cm^–1^ for the NH of residue 2 which forms a 7-cycle with its H-bond, 1535 ± 2 cm^–1^ for the NH:s of residues 1 and 4 which form longer cycles with their H-bonds, and 1505 cm^–1^ for the free NH of residue 3. In experiment, however, these bands are not resolved.
- Around 1440 cm^–1^ is a bump corresponding to CH_3_ scissoring. There is also a notable CH_2_ scissoring in the Phe residue which slightly mixes with NH movement and therefore has a greater activity, seen in the predicted spectra at 1462 cm^–1^.
- At 1368 cm^–1^ there is a band predicted to correspond to umbrella inversion of the acetate cap methyl group. The activity of this mode is amplified by the movement of nearby polar groups.
- At 1314 cm^–1^ there is a band corresponding to the CH rocking modes in the four chiral carbons. The modes are generally counterbalanced by movement of the adjacent NH group.
- The peaks at 1224 and 1266 cm^–1^ belong to the amide III band, meaning in-plane bending of NH counterbalanced by carbonyl movement. Because of the caps there are five such modes, of which two behave differently. The second amide III mode from the N-terminus has a lower frequency because of a 7-cycle H-bond, and is assigned to 1224 cm^–1^. Also, the mode at the C-terminus has a high frequency of 1291 cm^–1^, likely because its amide group is primary (NH_2_).
- At 1200 cm^–1^ lies a Phe CH_2_ twisting mode which also mixes in movement of the two closest NH groups.
- At 1160 cm^–1^ lies a delocalized mode involving the first two Ala residues, with intense movement in the first NH and its methyl group.

Beyond here, the vibrational modes are difficult to describe other than that they involve more than half of the molecule. Bands down to 850 cm^–1^ can be assigned vibrational modes simply by proximity. Below 850 cm^–1^, assignment is more difficult. One vibrational mode in this range is particularly localized:

- The intense predicted mode at 517 cm^–1^ corresponds to simple NH_2_ wagging. There is an experimental band at this frequency, but the intense band at 489 cm^–1^ is a candidate for assignment that cannot be overlooked. One known way of assigning this band would be to repeat the experiment with the nitrogen substituted for another isotope, which would noticeably shift the true NH_2_ wagging band.

**Figure 5:**
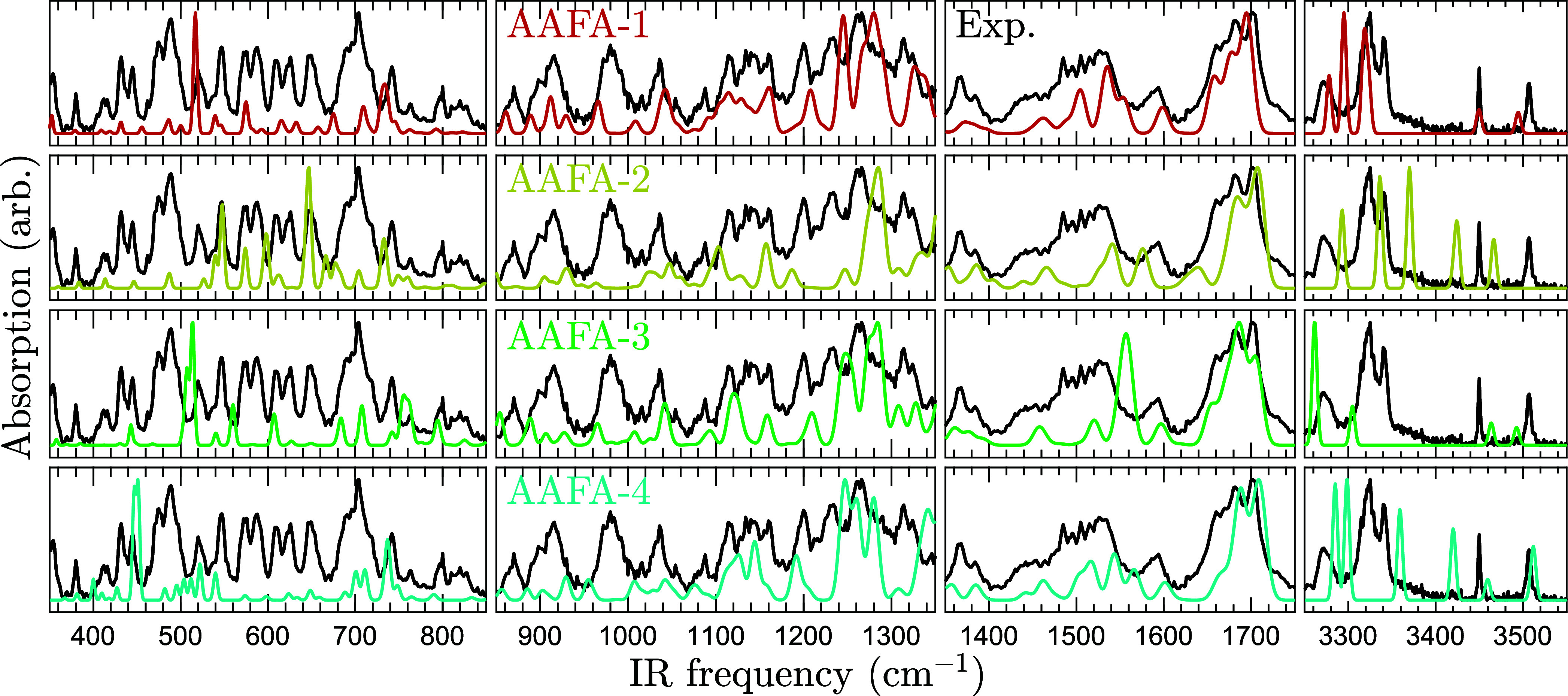
Comparison between experimental AAFA spectrum and theoretical predictions. The solid black curves repeatedly show the experimental absorption signal. The solid colored curves show the B3LYP/Jun-cc-pVTZ harmonic frequency predictions of the four most stable conformers. The theoretical spectra are scaled by 0.980 below 2000 cm^–1^ and 0.955 above.

To summarize the assignment: there is clear agreement between theory and experiment, and the IR bands above 850 cm^–1^ can be assigned vibrational modes by proximity.

The confirmed AAFA-1 conformer has a β-hairpin structure, which as stated previously is common to several tetrapeptides. The fact that AAFA does not form a helix should therefore be seen as a result of its size. For example, an α-helix is stabilized by *i* + 4 → *i* hydrogen bonds, which are possible in AAFA only thanks to the caps adding peptide links. Only for longer peptides can the α-helix pattern create enough hydrogen bonds to be stable. A 3_10_ structure would permit two *i* + 3 → *i* hydrogen bonds, but no conformer with only two hydrogen bonds has been found among all DFT-optimized structures.

#### 3.3.2 The Pentapeptide AAFAA

Figure 6 compares the theoretical spectra of AAFAA to experiment. Near the stretching band at 3510 cm^–1^, believed to correspond to an NH motion, a faint second feature is seen. A likely explanation is that another conformer was also ionized, though at a lesser rate. The theoretical predictions match the experiment poorly.

**Figure 6:**
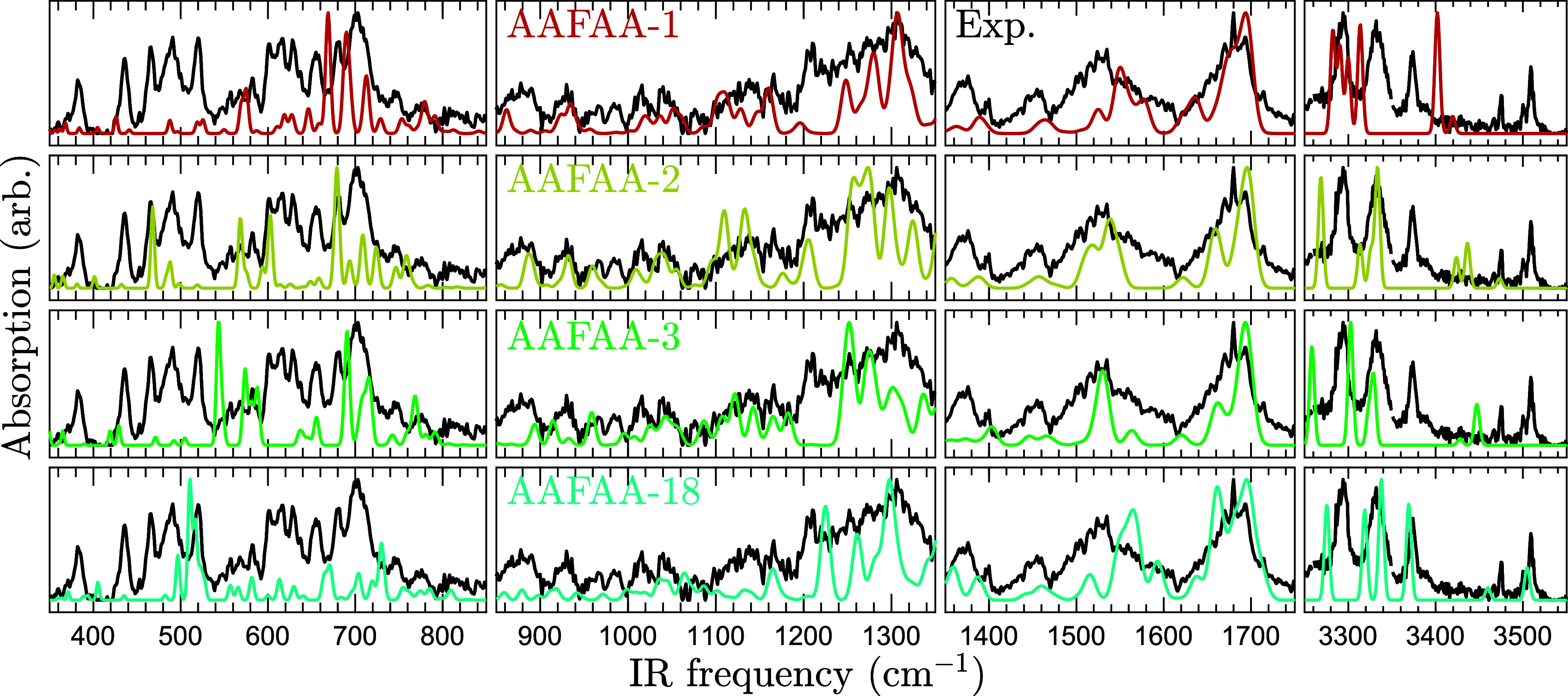
Comparison between experimental AAFAA spectrum and theoretical predictions. The solid black curves repeatedly show the experimental absorption signal. The solid colored curves show the B3LYP/Jun-cc-pVTZ harmonic frequency predictions of the four most stable conformers. The theoretical spectra are scaled by 0.980 below 2000 cm^–1^ and 0.955 above.

Out of all conformers that decently predict the two IR bands above 3400 cm^–1^, which correspond to primary amide stretching modes, the one with lowest energy is AAFAA-18. However, its energy is still too high, 23.82 kJ mol^–1^ ∼ 2865 K above AAFAA-1 according to CBS-4M. Furthermore, its spectrum does not match the experiment well outside of the amide A region, further indicating it is not the conformer seen in experiment. This negative result likely means that the true conformer of AAFAA has not been found.

Although the true structure of AAFAA was not found, the existence of AAFAA-18 gives a clue. The agreement between its primary amide modes and experiment reveals a plausible chemical environment for the amide group. In AAFAA-18, one hydrogen is free and the other is H-bonded to the carbonyl group of the second alanine. Since the vibrational frequencies in the stretching range are sensitive to the local chemical environment, it stands to reason that the primary amide of the true structure similarly has one free and one H-bonded hydrogen. Such knowledge could be applied as heuristic bound on interatomic distances in conformer searches.

### 3.4 Search Validity

Because the theoretical results concerning AAFAA are poor, it is appropriate to reflect on the validity of our methods.

The first generation of the conformational search started out with four structures, and generated 25,000 from each, finding similar and stable structures earlier. In the investigation of AAFA, the AAFA-1 conformer was generated early (before the 1000th) from every starting structure. This suggests that 25,000 was a sufficient number and that the choice of starting point was not critical.

On the flip side, the conformer of AAFAA seen in experiments has likely not been found despite the generation of more than 200,000 conformers. Furthermore, the first three generations of conformer searching contributed with distinct low-energy conformers, implying that each generation was insufficient on its own. In general, requiring consistent results is an intuitive condition of convergence. In the case of conformer searching, it is inefficient because of redundant optimizations in parallel searches. A better strategy would be to extend the search method (in this case, basin-hopping) to track not only the conformers found, but also the number of times they have been generated. If these numbers for the most stable conformers were small, it would be a sign of an insufficient search.

In summary, the message is mixed. The true conformer of AAFA was consistently found after 25,000 iterations, but the true conformer of AAFAA is still not yet found after more than 200,000. The latter result should be taken as a sobering reminder of the difficulty of predicting peptide folding.

### 3.5 Phenyl Group Effect

As part of the extended investigation of AAFAA, a conformer search of unphenylated capped pentaalanine (AAAAA) was carried out, and a phenyl group was then substituted onto the middle residue. This did not only produce conformers, but also allowed the role of the phenyl group to be studied.

Figure 7 shows the relative energies of AAAAA conformers before and after adding the phenyl group. All energies were computed with CBS-4M at 0 K on structures optimized with B3LYP/Jun-cc-pVTZ. Surprisingly, the addition of the phenyl group greatly affected the energy order; the most stable AAFAA conformer found this way came from AAAAA-6.

**Figure 7:**
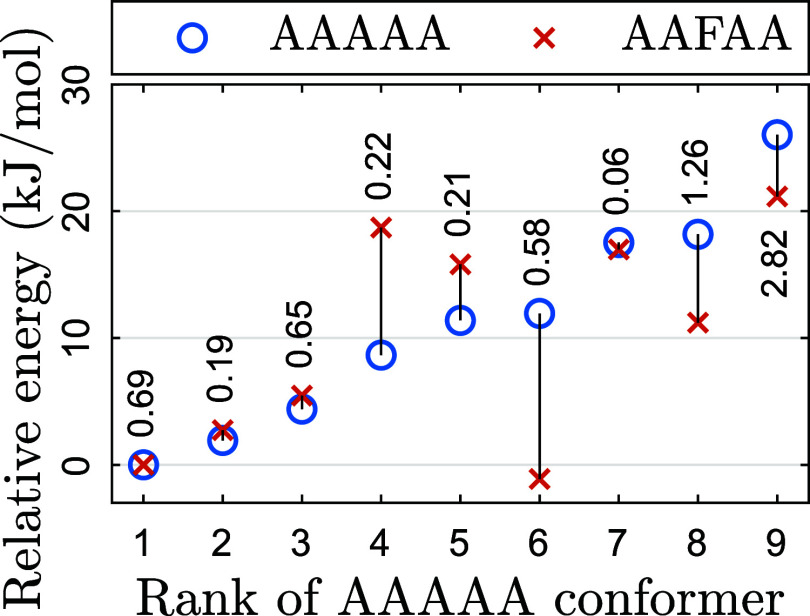
Relative energies of corresponding (blue circles) AAAAA and (orange crosses) AAFAA conformers, computed using CBS-4M (0 K). The RMSD between pairs expressed in Å is written in black text.

The optimized geometries were not significantly altered by the phenyl group. When comparing corresponding conformers, the median RMSD was 0.6 Å. These RMSDs between AAAAA and AAFAA conformers were computed after omitting the third side chain.

Because the phenyl group so greatly affected the energy order of the pentapeptide conformers, the analysis was repeated with the tetramers, this time by pruning the phenyl group of known stable conformers of AAFA. Figure 8 shows the result. While relative energies change, the dominant conformer remains.

**Figure 8:**
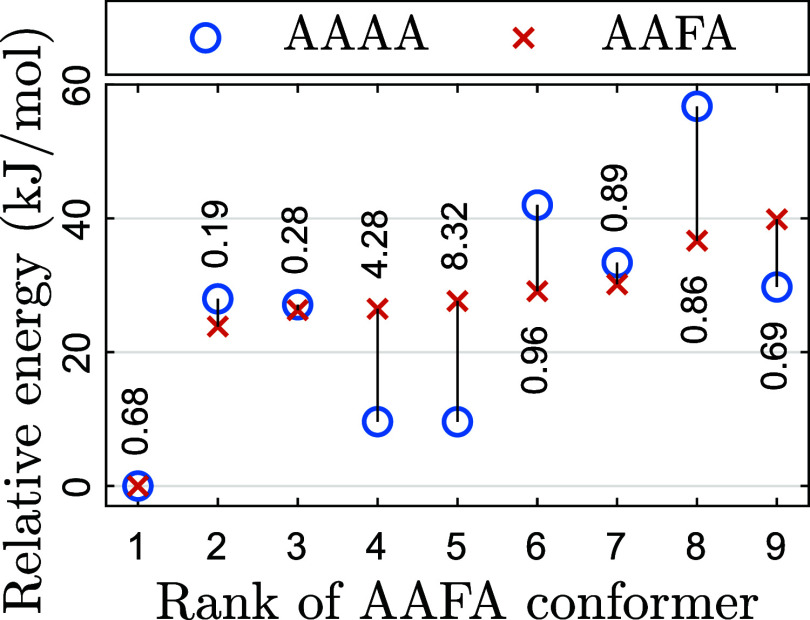
Analogous to Figure 7, but for the tetrapeptide AAFA.

The fact that adding a phenyl group changes which conformer has the lowest electronic energy, has interesting implications. IR–UV ion dip spectroscopy requires a chromophore, and therefore such studies of peptides most often substitute a phenyl group onto an alanine residue or cap. An implicit assumption of this strategy has been that the phenyl group does not alter the structure, an assumption which the present result challenges. It is plausible that other phenylated peptides adopt a different structure than their unphenylated counterparts. To study the latter, a chromophore-less technique such as the IRMPD–VUV method can be used.^32^

## 4 Conclusions

The IR–UV ion dip spectra of gas-phase AAFA and AAFAA were obtained in the frequency ranges 300–1900 and 3200–3600 cm^–1^. The AAFA peptide adopts a single dominant conformer with three REMPI resonances assigned to the fundamental transition and two vibrational overtones. The AAFAA peptide showed a broader REMPI spectrum, with some evidence for more than one conformer in the experiment, although only one IR–UV ion dip spectrum was obtained.

Theoretical investigations of the AAFA species were successful and yielded a remarkably stable conformer with a previously seen so-called β-hairpin structure.^17,20^ The predicted spectrum using B3LYP/Jun-cc-pVTZ matched the experiment well, and varying the functional did not improve the result. The active IR bands above 850 cm^–1^ can be assigned vibrational modes by proximity, and those modes above 1150 cm^–1^ have been explicitly described.

On the other hand, investigations into the structure of AAFAA gave a negative result. No remotely stable conformer found can explain the experimental spectrum. A likely explanation is that our conformational searches did not find the true conformer, despite running for more than 200,000 iterations. This is a sobering negative result, as conformer searches have been employed with far fewer iterations before.^2^

The addition of the phenyl group was found to significantly affect the energy ranking, but not the structure of conformers of the pentapeptide AAAAA and to a lesser extent AAAA. By contrast, the structure and spectrum of AAFA were similar to the previously studied tetrapeptides with different chromophore placement.^17,20^ This raises the question of whether the practice of phenyl substitution for IR–UV ion dip spectroscopy changes the conformational landscape.
